# Transcriptomic analysis reveals the mechanism underlying the anthocyanin changes in *Fragaria nilgerrensis* Schlecht. and its interspecific hybrids

**DOI:** 10.1186/s12870-023-04361-1

**Published:** 2023-07-11

**Authors:** Aihua Wang, Hongye Ma, Xingtao Zhang, Baohui Zhang, Fei Li

**Affiliations:** 1 School of Biological and Food Engineering, Engineering Research Center for Development and High Value Utilization of Genuine Medicinal Materials in North Anhui Province, Suzhou University, Suzhou, 234000 Anhui China; 2 Horticulture Institute (Guizhou Horticultural Engineering Technology Research Caenter), Guizhou Academy of Agricultural Sciences, Guiyang, 550006 China

**Keywords:** *Fragaria nilgerrensis* Schlecht, Anthocyanin biosynthesis, Interspecific hybrids, Pelargonidin derivatives, Red coloration

## Abstract

**Background:**

*Fragaria nilgerrensis* (FN) provides a rich source of genetic variations for strawberry germplasm innovation. The color of strawberry fruits is a key factor affecting consumer preferences. However, the genetic basis of the fruit color formation in *F. nilgerrensis* and its interspecific hybrids has rarely been researched.

**Results:**

In this study, the fruit transcriptomes and flavonoid contents of FN (white skin; control) and its interspecific hybrids BF1 and BF2 (pale red skin) were compared. A total of 31 flavonoids were identified. Notably, two pelargonidin derivatives (pelargonidin-3-O-glucoside and pelargonidin-3-O-rutinoside) were revealed as potential key pigments for the coloration of BF1 and BF2 fruits. Additionally, dihydroflavonol 4-reductase (*DFR*) (LOC101293459 and LOC101293749) and anthocyanidin 3-O-glucosyltransferase (*BZ1*) (LOC101300000), which are crucial structural genes in the anthocyanidin biosynthetic pathway, had significantly up-regulated expression levels in the two FN interspecific hybrids. Moreover, most of the genes encoding transcription factors (e.g., MYB, WRKY, TCP, bHLH, AP2, and WD40) related to anthocyanin accumulation were differentially expressed. We also identified two *DFR* genes (LOC101293749 and LOC101293459) that were significantly correlated with members in bHLH, MYB, WD40, AP2, and bZIP families. Two chalcone synthase (*CHS*) (LOC101298162 and LOC101298456) and a *BZ1* gene (LOC101300000) were highly correlated with members in bHLH, WD40 and AP2 families.

**Conclusions:**

Pelargonidin-3-O-glucoside and pelargonidin-3-O-rutinoside may be the key pigments contributing to the formation of pale red fruit skin. *DFR* and *BZ1* structural genes and some bHLH, MYB, WD40, AP2, and bZIP TF family members enhance the accumulation of two pelargonidin derivatives. This study provides important insights into the regulation of anthocyanidin biosynthesis in FN and its interspecific hybrids. The presented data may be relevant for improving strawberry fruit coloration via genetic engineering.

**Supplementary Information:**

The online version contains supplementary material available at 10.1186/s12870-023-04361-1.

## Background

Cultivated garden strawberry (*Fragaria* × *ananassa*) is one of the most economically and commercially important fruit species worldwide. Modern strawberry breeding has problems such as a narrow parental genetic background and a lack of phenotypic diversity present in most breeding programs [1]. Using wild strawberry germplasm resources to germplasm innovation is one of the ways to break through the bottleneck of traditional breeding [2]. *Fragaria nilgerrensis* (FN) is a wild diploid strawberry species endemic to east and southeast region in Asia and provides a rich source of genetic variations for strawberry improvement [3]. Strawberry is widely consumed not only for its enriched bioactive compounds but also for its attractive fruit color [4]. Anthocyanins are prominent plant pigments that belong to the flavonoid family and contribute to strawberry fruit coloration [5, 6]. These compounds are responsible for producing various colors (e.g., red, blue, and purple) in plants, but they also have beneficial effects on human health (e.g., protection against stress)[7]. Earlier research revealed that different types and levels of anthocyanins bring us different colored strawberry [4]. The hybridization between *F. nilgerrensis* (white fruits) and *F.* × *ananassa* (red fruits) resulted in an interspecific decaploid hybrid (‘Tokun’) with a pink fruit color [8], suggesting that clarifying the genetic basis of anthocyanin accumulation may lead to critical insights into the metabolic mechanism underlying strawberry fruit color formation, with implications for improving fruit quality.

Nearly 700 different anthocyanins have been identified so far [4]. In straberry, pelargonidin-3-glucoside is the dominant anthocyanin in strawberries regardless of genetic and environmental factors, followed by derivatives of pelargonidin and cyanidin, but in considerably smaller amounts [9]. The regulation of the anthocyanin biosynthetic pathway has been widely established in plants [10, 11]. The major pathways involved in anthocyanin synthesis include the pentose phosphate pathway, shikimate pathway, and phenylpropanoid and flavonoid biosynthetic pathways [12]. Most structural genes encoding enzymes involved in anthocyanin biosynthesis have been isolated and characterized [13], such as phenylalanine ammonialyase (PAL), cinnamate-4-hydroxylase (C4H), chalcone isomerase (CHI), chalcone synthase (CHS) and anthocyanidin synthase (ANS), leading to different intermediates and different flavonoid classes are well known [14, 15]. However, most of these previous studies mainly focused on leaf and petal colors [16]. The genetic mechanism mediating the fruit coloration of *F. nilgerrensis* and its interspecific hybrids has rarely been researched prior to this study. Anthocyanin biosynthesis is a complex biological process regulated by multiple enzymes and transcription factors (TFs) [17], as well as being influenced by genetic, developmental, and environmental factors [18]. Thus, the mechanism regulating changes in anthocyanin compositions and contents in FN and its interspecific hybrids will need to be thoroughly investigated.

The structural genes related to enzyme reactions in anthocyanin biosynthesis pathway are largely regulated byTFs. In all species studied to date, MYB TFs, basic helix-loop-helix (bHLH) TFs, and WD-repeat proteins are the most extensively investigated types [19]. In strawberry, these proteins combine to form a ternary MYB–bHLH–WD40 complex that regulates proanthocyanidin accumulation [20], but they can also function independently. For example, MYB TFs control the red coloration of octoploid strawberry fruits [21]. Another MYB TF, MYB10, regulates the flavonoid/phenylpropanoid metabolism in *F.* × *ananassa* fruits during ripening [22]. Additionally, bHLH TFs have been linked with the development of white-fleshed mutant strawberry fruits [23]. More recently, many other TF families have been demonstrated to be involved in anthocyanin modulation. The FvTCP9 TF reportedly promotes strawberry fruit ripening by controlling the biosynthesis of abscisic acid and anthocyanins [24], while MdWRKY72 promotes anthocyanin synthesis in apple [25]. In addition, the newly discovered TF complexes HY5–bHLH9 [26] and MdNAC42–MdMYB10 were confirmed as anthocyanin biosynthesis regulators [27]. These studies revealed the existence of complex regulatory networks. However, there is limited information on the role of these transcription factors in strawberry anthocyanin regulation, and more novel transcription factors need to be identified and characterized..

To elucidate the mechanism underlying the changes in anthocyanin contents in FN and its interspecific hybrids, the transcriptome analysis of FN fruits (white skin) as well as the fruits from its interspecific hybrids BF1 and BF2 (pale red skin) were conducted in the present study. Their flavonoids were also analyzed using an ultra-high-performance liquid chromatography–electrospray ionization–tandem mass spectrometry (UPLC-ESI–MS/MS) system. The identification of anthocyanin compounds and analyses of differentially expressed genes (DEGs) (structural DEGs and TFs related to anthocyanin biosynthesis, and correlation between them) in FN and its interspecific hybrids with different phenotypes will expand the current understanding of strawberry anthocyanin biosynthesis and shed light on the potential genetic mechanism of fruit colour formation in *F. nilgerrensis* and its interspecific hybrids. The results of the present study may serve as a solid foundation for breeding and utilization of wild strawberry resources in the future.

## Results

### Differences in the phenotypes and colors between *F. nilgerrensis* and its interspecific hybrids

**Fig. 1:**
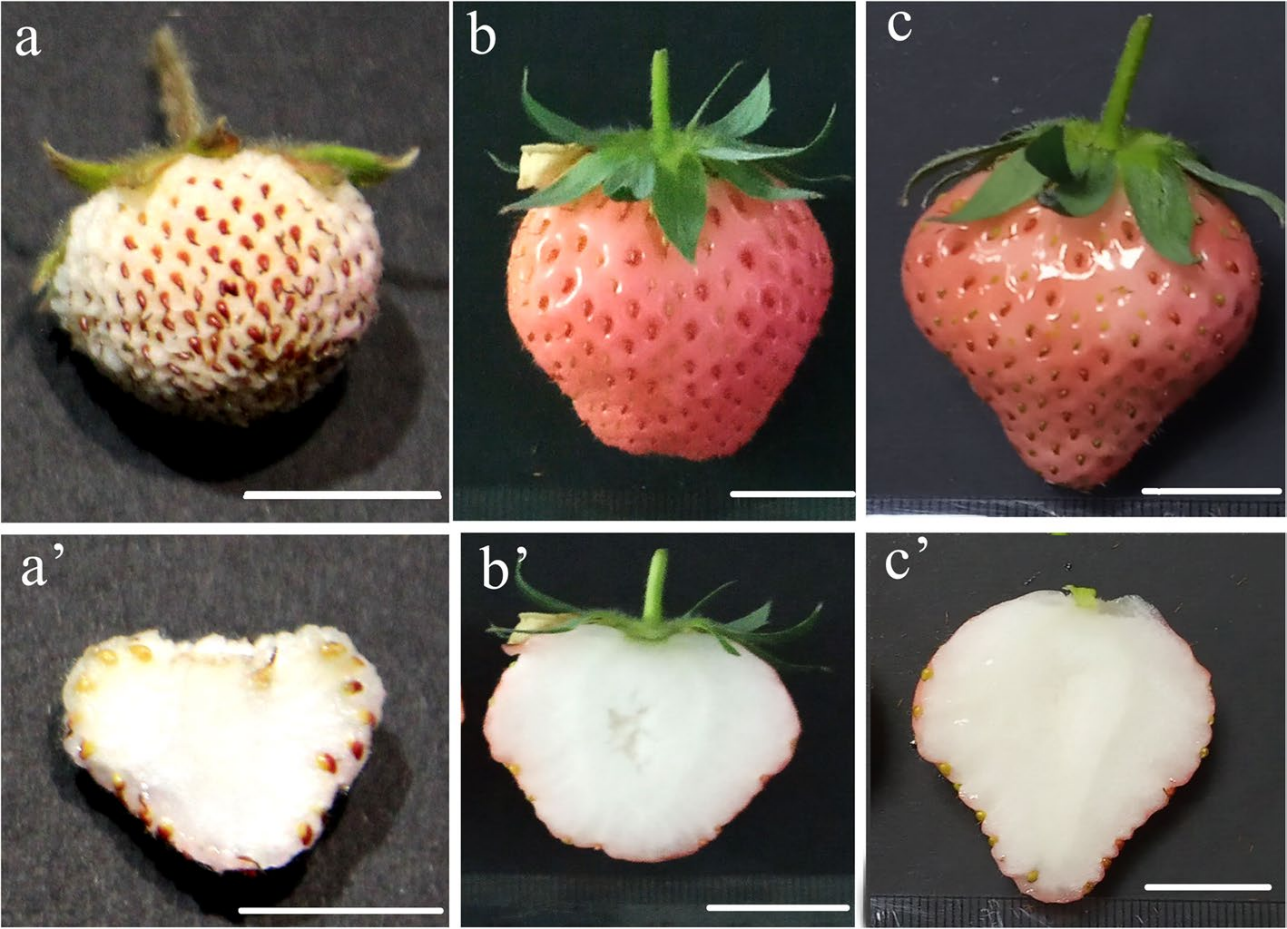
Fruits of *F. nilgerrensis Schlecht*. (FN, a and a’) and its interspecific hybrids BF1(b and b’) and BF2 (c and c’). The scale bar represents 1 cm

The comparison of the fruit skin color of FN and its two interspecific hybrids BF1 and BF2 revealed that the a* (green–red coordinate) and C* (color saturation) values were significantly lower for FN than for BF1 and BF2, whereas the L* (lightness) value was significantly higher for FN than for BF1 and BF2 (Fig. 1). The a* values for FN, BF1, and BF2 were 3.76 ± 0.67, 22.75 ± 1.4, and 29.55 ± 2.29, respectively (Table 1). These results were consistent with the observed phenotypic difference in the fruit skin color between the interspecific hybrids (uniformly pale red) and FN (white).

**Table 1:** Color values in *F. nilgerrensis* Schlecht. (FN) and its interspecific hybrids BF1 and BF2

Sample	L*	a*	b*	C*
NF	51.81 ± 2.17a	3.76 ± 0.67c	12.12 ± 1.64a	12.70 ± 1.63c
BF1	31.41 ± 1.49b	22.75 ± 1.40b	7.42 ± 1.13ab	23.97 ± 1.06b
BF2	31.85 ± 0.65b	29.55 ± 2.29a	8.85 ± 0.69b	30.86 ± 2.03a

L* ,a*, b*, C* represent lightness

### Metabolomic profiling of *F. nilgerrensis* and its interspecific hybrids

The flavonoid contents of the FN, BF1, and BF2 fruits were analyzed using the UPLC-ESI–MS/MS system, which detected substantial differences in the anthocyanin profiles of FN and its interspecific hybrids. Using a local metabolite database, a total of 108 metabolites were identified, including 17 cyanidins, 16 delphinidins, 13 malvidins, 19 pelargonidins, 28 pelargonidins, 6 procyanidins, and 9 flavonoids (Table S1).

A total of 31 DAMs were detected from the NF *vs* BF1 and NF *vs* BF2 comparisons (Table 2), of which 17 DAMs were co-up-regulated and 14 DAMs were co-down-regulated. The co-up-regulated DAMs were mainly pelargonidins and peonidins, including pelargonidin-3-O-galactoside, pelargonidin-3-O-glucoside, pelargonidin-3-O-rutinoside, pelargonidin-3-O-arabinoside, pelargonidin-3,5-O-diglucoside, pelargonidin-3-O-(6-O-malonyl-beta-D-glucoside), peonidin-3-O-glucoside, peonidin-3-O-(6-O-p-coumaroyl)-glucoside, and peonidin-3-O-rutinoside, which were significantly more abundant in BF1 and BF2 than in FN. In particular, two pelargonidins, namely pelargonidin-3-O-sophoroside-5-O-(malonyl)-glucoside and pelargonidin-3-O-sophoroside, and two peonidins, namely peonidin-3-O-(6-O-malonyl-beta-D-glucoside) and peonidin-3-O-sambubioside, were highly abundant in BF1 and BF2, but were undetectable in FN. Additionally, the cyanidin, delphinidin, procyanidin, and flavonoid contents were lower in BF1 and BF2 than in FN. These results suggested that anthocyanins may be crucial for the formation of strawberry fruits with a pale red skin. Moreover, the considerable accumulation of pelargonidin and peonidin derivatives may be associated with the color-related changes in BF1 and BF2 fruits.

**Table 2:** Differentially accumulated flavonoids in *F. nilgerrensis* Schlecht. (FN) and its interspecific hybrids BF1 and BF2

Class	Compounds	Content/μg·g^−1^			BF1 vs FN	BF1 vs FN	BF2 vs FN	BF2 vs FN
		FN	BF1	BF2	Fold change	*P-value*	Fold change	*P-value*
Pelargonidin	Pelargonidin-3-O-galactoside	3.85E-01	1.03E + 01	1.24E + 01	26.86	9.04E-05	32.23	1.87E-02
	Pelargonidin-3-O-glucoside	2.46E + 01	3.88E + 02	4.14E + 02	15.78	1.31E-03	16.85	1.27E-04
	Pelargonidin-3-O-rutinoside	1.81E + 00	8.92E + 01	9.12E + 01	49.33	8.00E-04	50.47	1.00E-04
	Pelargonidin-3-O-arabinoside	7.30E-03	4.13E-01	4.40E-01	56.76	5.32E-04	60.50	1.00E-04
	Pelargonidin-3-O-(6-O-p-coumaroyl)-glucoside	5.00E-04	2.16E-02	2.29E-02	42.27	1.50E-03	44.73	7.50E-03
	Pelargonidin-3,5-O-diglucoside	1.50E-02	6.69E-01	7.50E-01	44.65	5.00E-04	50.07	1.00E-04
	Pelargonidin-3-O-(6-O-malonyl-beta-D-glucoside)	4.81E-01	6.32E + 00	6.93E + 00	13.12	1.00E-04	14.39	2.50E-03
	Pelargonidin-3-O-sophoroside-5-O-(malonyl)-glucoside	0.00E + 00	1.23E-02	1.33E-02	inf	1.10E-03	inf	2.00E-04
	Pelargonidin-3-O-sambubioside	1.26E-01	0.00E + 00	0.00E + 00	-inf	8.00E-04	-inf	8.00E-04
	Pelargonidin-3-O-sophorosid	0.00E + 00	1.66E-01	1.69E-01	inf	5.00E-04	inf	3.00E-04
Peonidin	Peonidin-3-O-(6-O-malonyl-beta-D-glucoside)	0.00E + 00	9.86E-02	1.27E-01	inf	1.00E-04	inf	1.10E-03
	Peonidin-3-O-glucoside	1.43E-02	2.63E + 00	3.12E + 00	184.04	2.50E-03	218.63	9.70E-03
	Peonidin-3-O-(6-O-p-coumaroyl)-glucoside	2.40E-03	1.76E-02	1.79E-02	7.27	2.00E-04	7.40	5.00E-04
	Peonidin-3-O-rutinoside	1.90E-02	1.53E + 00	1.66E + 00	80.74	1.30E-03	87.60	5.00E-04
	Peonidin-3-O-sambubioside	0.00E + 00	1.40E-02	1.75E-02	inf	4.00E-04	inf	9.70E-03
Malvidin	Malvidin-3-O-glucoside	0.00E + 00	3.30E-03	2.50E-03	inf	3.50E-03	inf	1.92E-02
Petunidin	Petunidin-3-O-glucoside	0.00E + 00	2.50E-03	2.80E-03	inf	6.00E-04	inf	1.15E-02
Cyanidin	Cyanidin-3-(6-O-p-caffeoyl)-glucoside	1.96E-02	0.00E + 00	0.00E + 00	-inf	3.00E-04	-inf	3.00E-04
	Cyanidin-3-O-(6-O-p-coumaroyl)-glucoside	2.04E-01	4.80E-03	3.30E-03	0.02	6.00E-04	0.016	5.00E-04
	Cyanidin-3-O-sambubioside	1.95E-01	3.10E-02	5.52E-02	0.16	1.90E-03	0.28	9.60E-03
Delphinidin	Delphinidin-3,5-O-diglucoside	5.06E-02	2.20E-03	2.70E-03	0.04	1.80E-03	0.05	2.00E-03
	Delphinidin-3-O-rutinoside-5-O-glucoside	3.48E-02	9.20E-03	9.60E-03	0.27	1.60E-03	0.28	1.30E-03
Procyanidin	Procyanidin B2	6.00E + 00	1.08E + 00	1.29E + 00	0.18	9.00E-04	0.21	1.20E-03
	Procyanidin A1	1.72E-02	0.00E + 00	0.00E + 00	-inf	6.90E-03	-inf	6.90E-03
Flavonoid	Naringenin-7-O-glucoside	6.84E + 00	1.16E + 00	1.60E + 00	0.17	1.00E-04	0.23	1.00E-04
	Dihydrokaempferol	2.81E-02	3.35E-01	4.52E-01	11.93	1.01E-02	16.10	5.00E-04
	Afzelin	4.57E-01	2.10E-01	1.98E-01	0.46	2.07E-02	0.43	2.21E-02
	Naringenin	1.16E-02	2.70E-03	5.70E-03	0.23	1.05E-02	0.47	4.83E-02
	Kaempferol-3-O-rutinoside	1.17E + 02	2.93E + 00	2.66E + 00	0.03	1.00E-04	0.02	1.00E-04
	Quercetin-3-O-glucoside	1.41E + 02	6.87E + 00	7.57E + 00	0.05	1.00E-04	0.05	1.00E-04
	Rutin	1.15E + 02	2.07E + 00	1.86E + 00	0.02	1.00E-04	0.02	1.00E-04

*Note*: 0.00E + 00 represents a barely detectable level. inf means infinity. -inf means infinitesimal

### Illumina sequencing and assembly

Transcriptome sequencing data were used to elucidate the molecular mechanism of flavonoid changes underlying the FN and its two interspecific hybrids. Three biological replicates of ripe fruits were analyzed by Illumina RNA-seq, which generated more than 42.83 million raw reads per sample. After the quality control, 41.41–46.82 million clean reads were yielded with more than 97.52% of Q20, and 32.86 to 37.47 million clean reads were mapped to the *Fragaria vesca* genome (mapping rate of 74.12%–80.02%) (Table 3).

**Table 3:** Sequencing quality for the fruit samples from *F. nilgerrensis* Schlecht. (FN) and its interspecific hybrids BF1 and BF2

Sample	Raw Reads(M)	Clean Reads(M)	Q20(%)	Reads mapped(M)	Mapping ratio (%)
NF -1	47.29	44.36	97.89	32.88	74.12
NF -2	46.75	43.77	98.18	33.57	76.70
NF -3	47.58	44.09	97.52	33.12	75.12
BF1 -1	42.83	41.41	98.16	32.86	79.35
BF1 -2	47.94	46.25	98.05	36.95	79.89
BF1 -3	47.90	46.82	98.01	37.47	80.02
BF2 -1	46.21	44.22	98.12	34.72	78.51
BF2 -2	46.12	43.44	98.09	34.24	78.82
BF2 -3	47.69	45.50	98.06	35.96	79.03

**Fig. 2:**
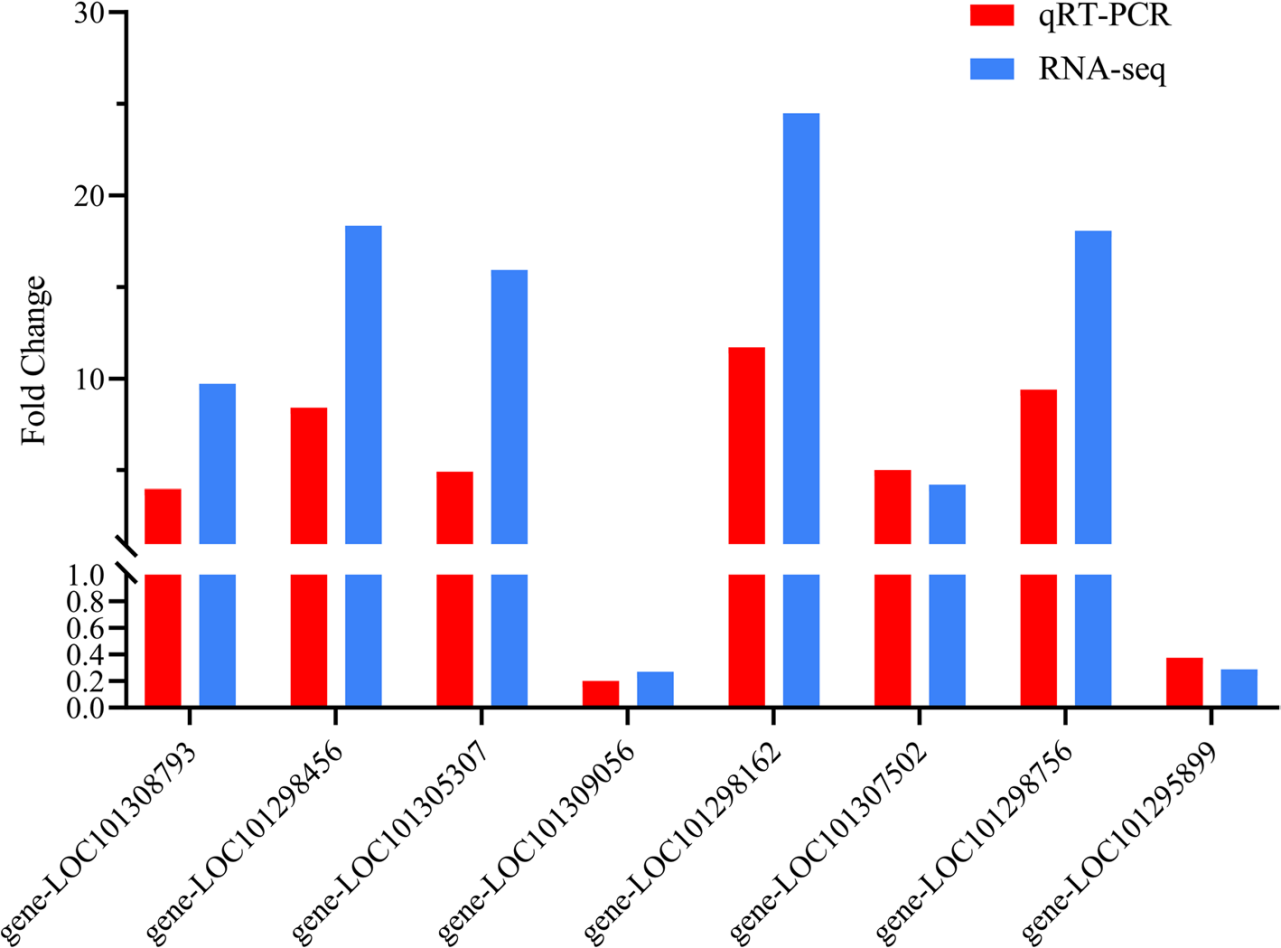
Validation of 8 selected DEGs via qRT-PCR

To verify the reliability of the RNA-seq data, the expression levels of eight DEGs involved in anthocyanin biosynthesis were analyzed in a qRT-PCR assay. The relative changes in the expression of these eight genes (Fig. 2) indicated the qRT-PCR results were in accordance with the RNA-seq data.

### Identification and functional analysis of DEGs

**Fig. 3:**
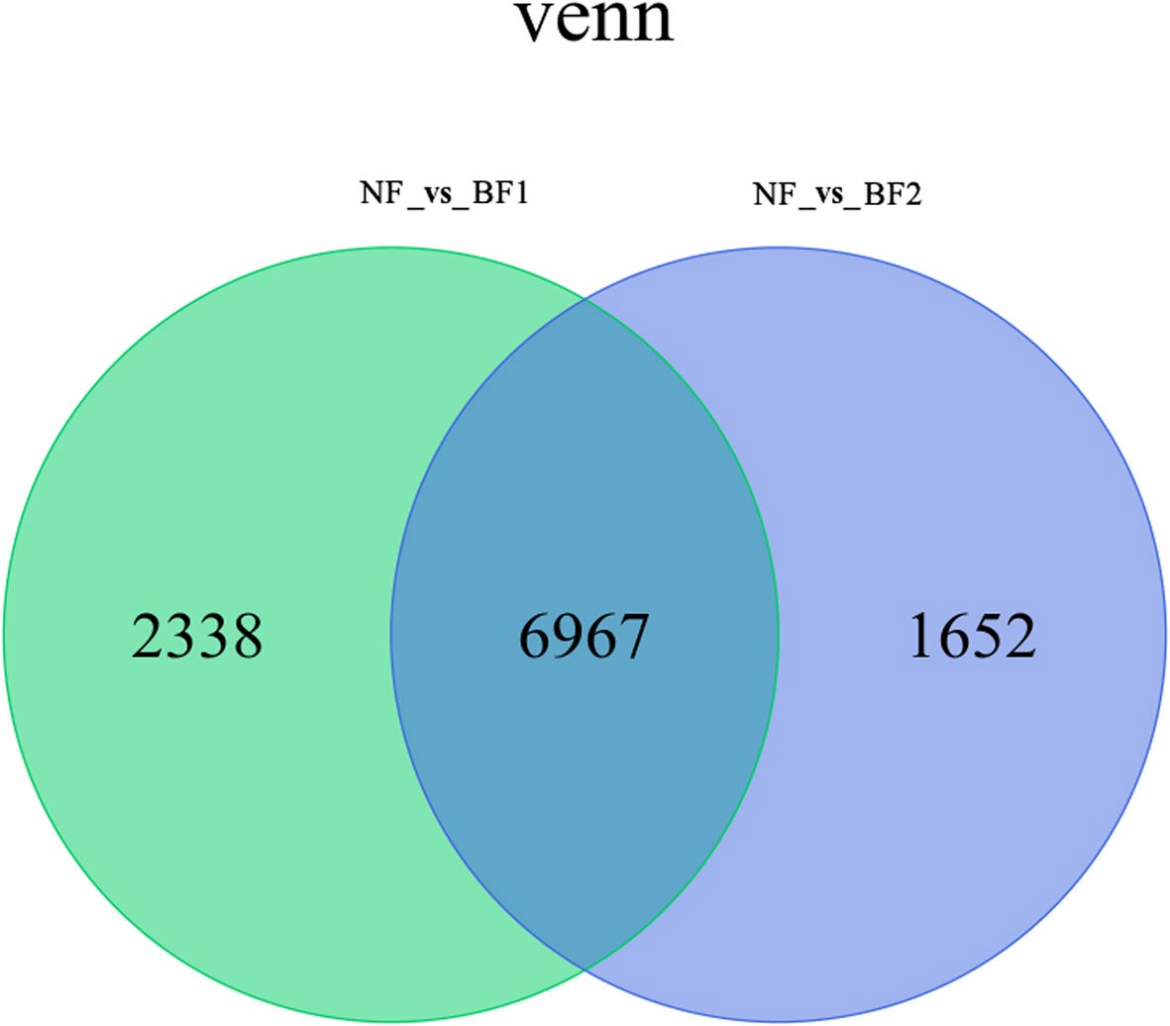
Venn diagram analysis of differentially expressed genes

To identify DEGs, the RNA-seq data were compared as follows: NF *vs* BF1 and NF *vs* BF2. A total of 10,957 DEGs were identified from these two comparisons, of which 6,967 were common to both comparisons, 2,338 were specific to the NF *vs* BF1 comparison, and 1,652 were exclusive to the NF *vs* BF1 comparison. A Venn diagram was drawn to illustrate the number of DEGs in both comparisons (Fig. 3). These results implied the co-differentially expressed genes in the two comparison groups may be related to the anthocyanins changes in FN and its interspecific hybrids.

**Fig. 4:**
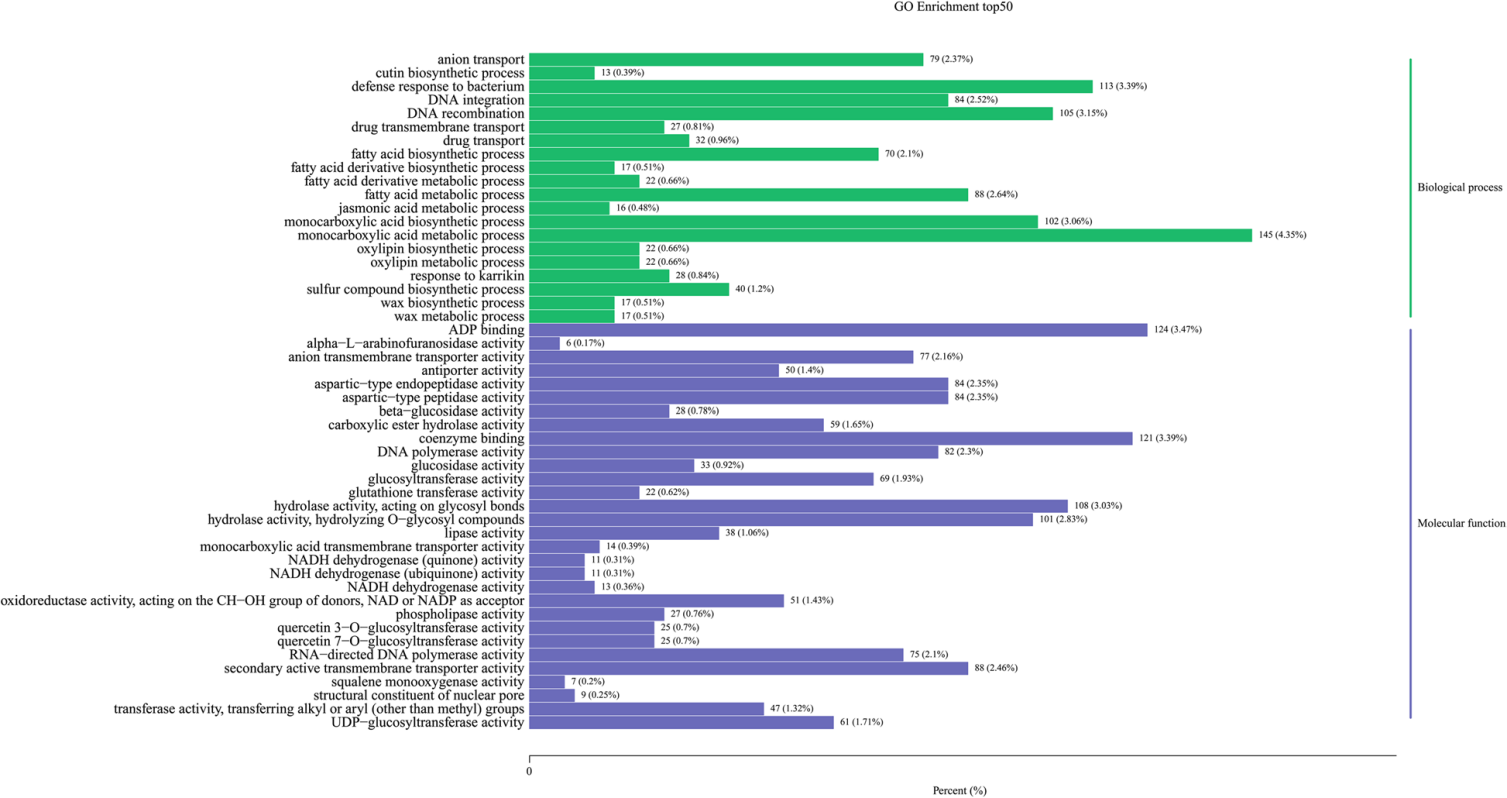
GO analysis of co-differentially expressed genes

To functionally characterize the DEGs, the significant DEGs detected in both comparisons were included in GO and KEGG enrichment analyses. The 50 most enriched GO terms from the biological process and molecular function categories were determined. The main biological process GO terms assigned to the co-DEGs were “monocarboxylic acid metabolic process,” “defense response to bacterium,” “DNA recombination,” and “monocarboxylic acid biosynthetic process.” The most enriched molecular function GO terms among the co-DEGs were “ADP binding,” “coenzyme binding,” “hydrolase activity, acting on glycosyl bonds,” and “secondary active transmembrane transporter activity.” The DEGs related to glucosidase activity (e.g., beta-glucosidase activity) and glucosyltransferase activity (e.g., UDP–glucosyltransferase activity) may be associated with anthocyanin synthesis in FN and its interspecific hybrids (Fig. 4).

**Fig. 5:**
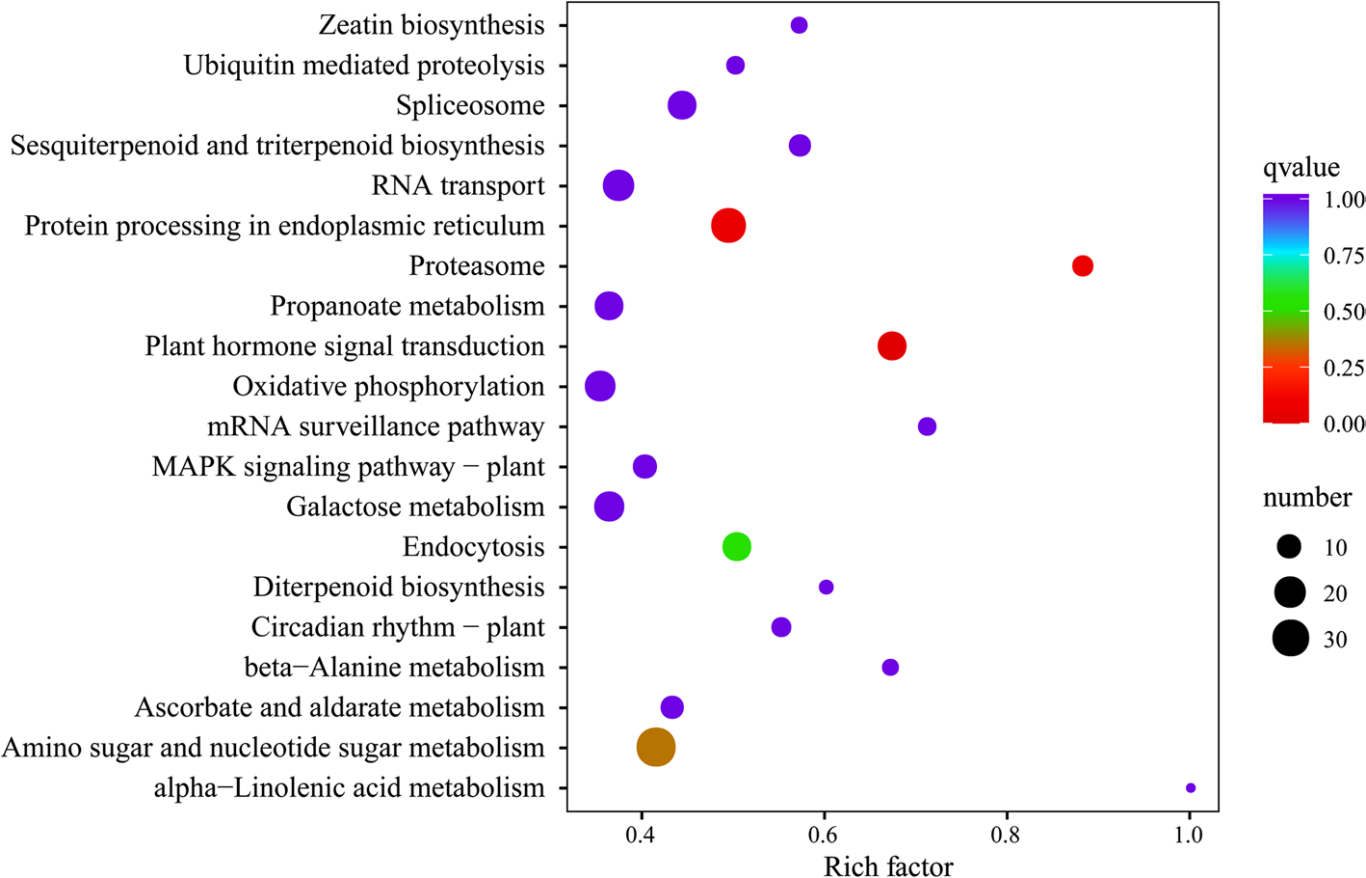
KEGG analysis of co-differentially expressed genes

A total of 6,967 co-significant DEGs were mapped to 109 KEGG pathways (Table S2). The 20 most enriched KEGG pathways among the co-DEGs revealed by the NF *vs* BF1 and NF *vs* BF2 comparisons were determined (Fig. 5). There were two pathways with a rich factor greater than 0.8, namely alpha–linolenic acid metabolism (ko00592) and proteasome (ko03050). The three pathways with the highest q-values were plant hormone signal transduction (ko04075), protein processing in endoplasmic reticulum (ko04141), and proteasome (ko03050).

### Expression patterns of structural DEGs related to anthocyanin biosynthesis

**Fig. 6:**
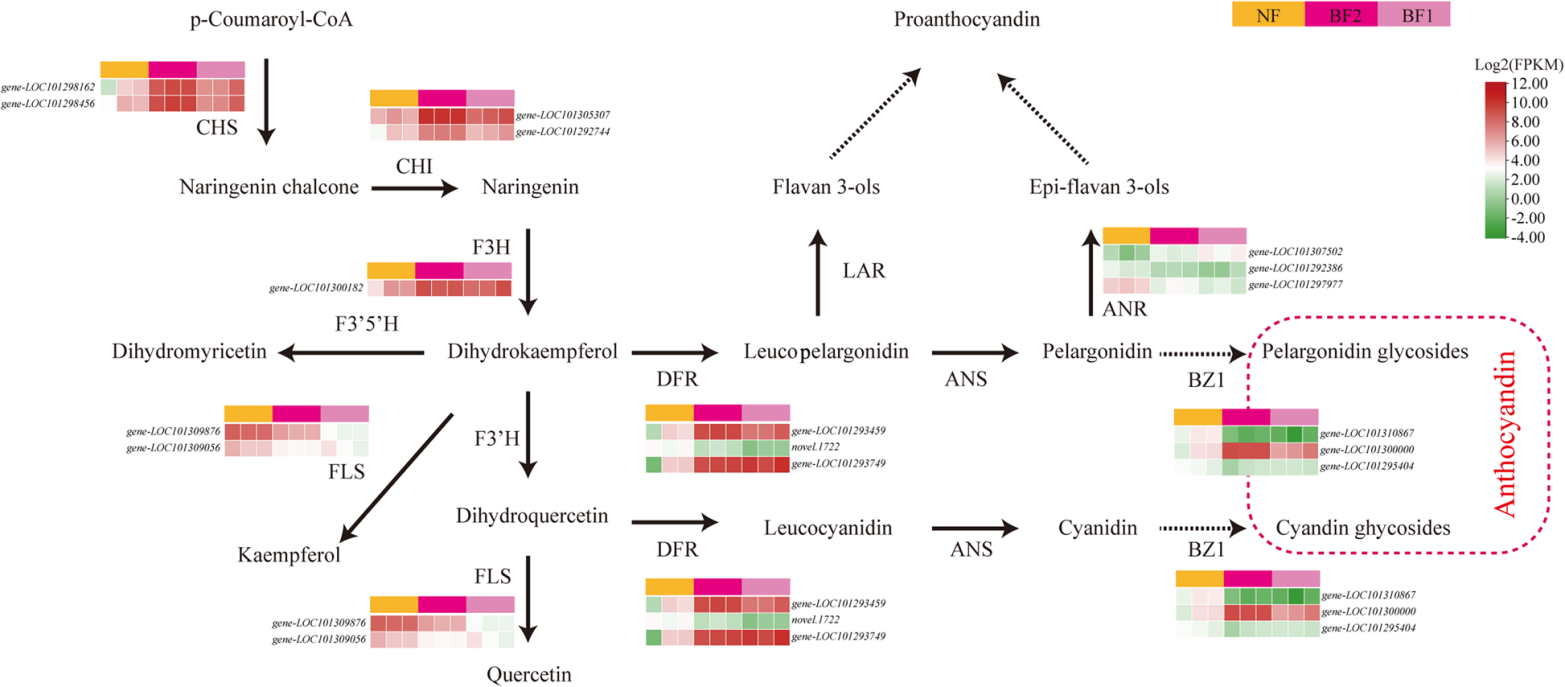
Expression patterns of structural DEGs related to anthocyanin biosynthesis in *F. nilgerrensis* and its interspecific hybrids. The level of gene expression was measured in *F. nilgerrensis* Schlecht. (FN) and its interspecific hybrids BF1 and BF2. Red indicatesa high level of expression at different samples. The the red dashed box represents the downstream metabolites of *BZ1* gene. The abbreviations of each gene that encodes enzymes and compounds are provided in abbreviations. The levels of gene expression in these biosynthetic pathways are shown in Table S3

Sixteen structural DEGs were predicted to be associated with anthocyanin biosynthesis. On the basis of the KEGG database, the flavonoid biosynthetic and anthocyanin biosynthetic pathways in multiple species [28, 29], and the relative expression levels of these 16 genes, we constructed an improved diagram of the anthocyanin biosynthetic pathway (Fig. 6). These 16 DEGs encoded two flavonol synthases (FLSs), two chalcone synthases (CHSs), one naringenin 3-dioxygenase (F3H), three dihydroflavonol 4-reductases (DFRs), two chalcone isomerases (CHIs), three anthocyanidin reductases (ANRs), and three anthocyanidin 3-O-glucosyltransferases (BZ1s) (Table S3, Fig. 6). Among these genes, the *CHS*, *F3H*, and *CHI* expression levels were up-regulated, whereas *FLS* expression was down-regulated. Interestingly, the *BZ1*, *ANR*, and *DFR* expression trends varied between the comparisons. More specifically, *BZ1* (LOC101300000) expression was up-regulated 7.76- and 36.42-fold, *ANR* (LOC101307502) expression was up-regulated 9.42- and 4.22-fold, and *DFR* (LOC101293459/LOC101293749) expression was up-regulated 15.27-/37.82- and 43.38-/31.38-fold in the NF *vs* BF1 and NF *vs* BF2 comparisons, respectively (Table S3, Fig. 6). Therefore, these candidate genes will need to be further analyzed to determine how they influence anthocyanin biosynthesis in FN and its interspecific hybrids.

### Candidate transcription factor genes involved in anthocyanin biosynthesis

**Fig. 7:**
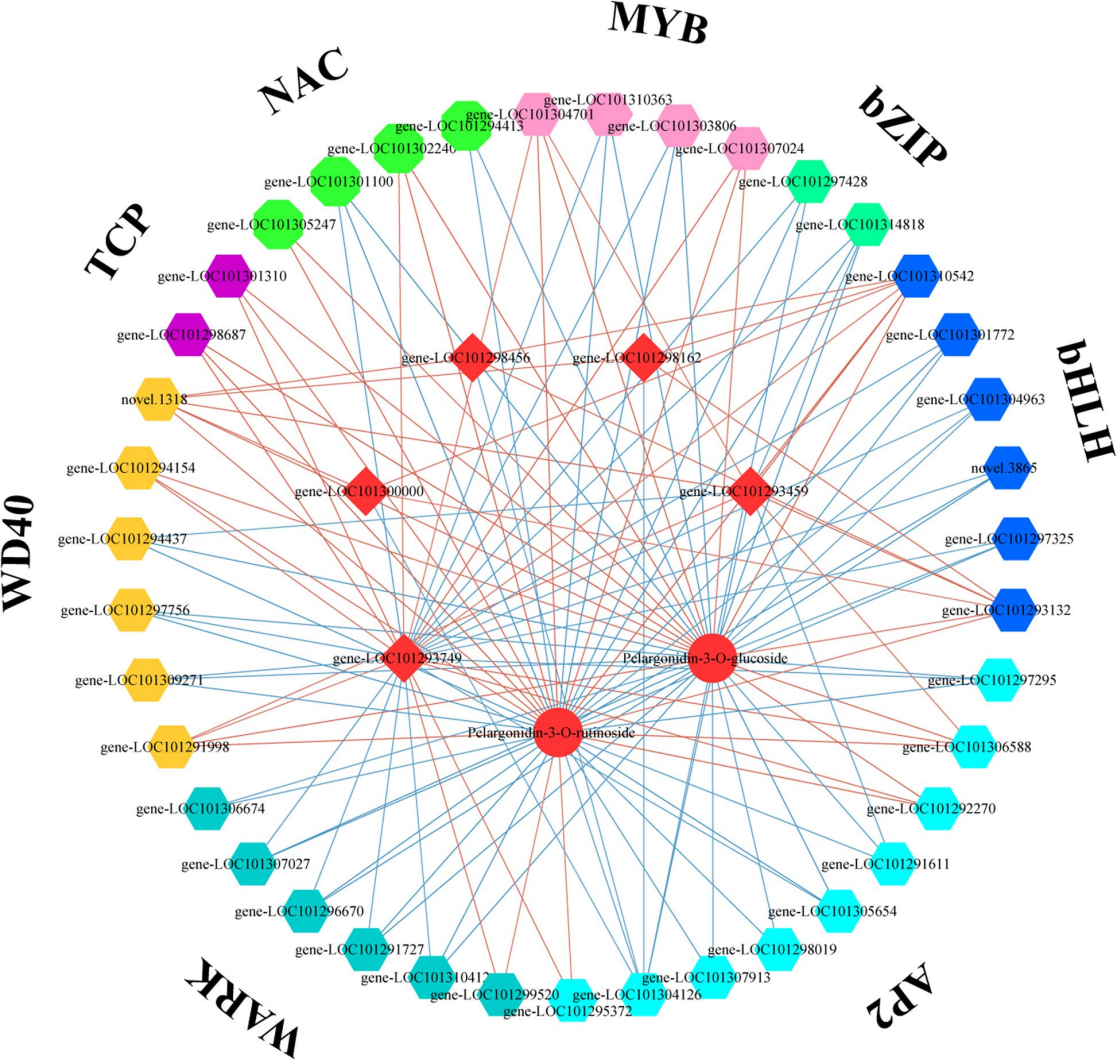
Correlation analysis between 39 transcription factor genes with 5 anthocyanin biosynthetic related genes and 2 key anthocyanins. The color filled hexagons represent different transcription factors, the red filled spheres represent geranidin-3-o-glucoside and geranidin-3-o-rubutin, and the red filled squares represent the structural genes associated with anthocyanin biosynthetic pathways. The expression correlations are shown with colored lines, and the red line indicates a positive correlation, and the blue line indicates a negative correlation

Transcription factors play an important role in anthocyanin synthesis. To identify the TFs involved in regulating the anthocyanin changes in FN and its interspecific hybrids, the correlations between 39 TF DEGs (Table S4) and 16 structural genes as well as 16 jointly up-regulated anthocyanins were analyzed (Tables S5, S6). We focused on TFs that are highly correlated with high content and co-upregulated geranidin-3-o-glucoside, geranidin-3-o-rubutin as well as the *CHS*, *DFR*, and *BZ1* genes. Both geranidin-3-O-glucoside and geranidin-3-O-rubutin were significantly correlated with bHLH, MYB, WRKY, NAC, WD40, TCP, AP2, and bZIP TF family members. Transcription factor genes that were highly correlated with *CHS* (LOC101298162 and LOC101298456) and *BZ1* (LOC101300000) included *bHLH* (LOC101293132 and LOC101310542), *WD40* (novel.1318), and *AP2* (LOC101304126). Transcription factor genes that were highly correlated with *DFR* (LOC101293459) included *MYB* (LOC101304701), *bHLH* (LOC101293132 and LOC101310542), *WD40* (novel.1318, LOC101294437, and LOC101291998), *AP2* (LOC101291611, LOC101304126, and LOC101306588), and *bZIP* (LOC101314818). Another *DFR* gene (LOC101293749) was significantly correlated with bHLH, MYB, WRKY, NAC, WD40, TCP, AP2, and bZIP TF family members (Fig. 7).

## Discussion

### Effects of anthocyanins on the fruit colors of *F. nilgerrensis* and its interspecific hybrids

Fruit colors affect consumer preferences, making them critical characteristics influencing the commercial value and aesthetic value of strawberry cultivars. Strawberry fruit color formation involves the accumulation of anthocyanins, which are major polyphenolic compounds in strawberry. Different anthocyanin types and contents are responsible for the diversity in fruit colors among strawberry cultivars (e.g., orange to extremely dark red) [30]. Pelargonidin and cyanidin derivatives are the most representative and important anthocyanins because their presence results in the production of red strawberry fruits [31]. To explore the molecular mechanism underlying strawberry fruit coloration, the anthocyanin components and contents were compared between mature FN fruits (white skin) and mature fruits (pale red skin) from the interspecific hybrids BF1 and BF2. The results showed that 17 DAMs (primarily pelargonidin and peonidin derivatives) were co-up-regulated (Table 2). Inconsistent with the findings of previous studies, the abundance of cyanidin derivatives, which produce a magenta fruit skin, was co-down-regulated, indicating that cyanidin derivatives may be lost during the fruit color formation of the FN interspecific hybrids [32].

In addition, the color red is closely related to the accumulation of anthocyanins during fruit maturity [33] and the anthocyanin concentration is an important index to measure fruit colors. Hence, the two pelargonidin derivatives that were detected at high concentrations (pelargonidin-3-O-glucoside and pelargonidin-3-O-rutinoside) may be the key pigments in the fruit coloration process of FN interspecific hybrids. Earlier research confirmed that strawberry fruit colors are related to changes in anthocyanin compositions [34]. Moreover, the production of peonidin derivatives may promote the formation of red fruits in FN progenies to some extent, which has rarely been demonstrated in previous studies.

### Major anthocyanin structural genes affect the fruit colors of *F. nilgerrensis* and its interspecific hybrids

The anthocyanin biosynthetic pathway involves many enzymes encoded by genes mainly from two classes. The early biosynthetic genes (e.g., *CHS*, *CHI*, and *F3H*) mediate the production of flavonoids, while the late biosynthetic genes (e.g., *DFR* and *ANS*) are associated with the production of anthocyanins [35–38]. We revealed that most of the structural genes in the flavonoid and anthocyanidin biosynthetic pathways were highly expressed in the FN interspecific hybrids, resulting in the formation of pale red fruit skin (Fig. 6). We identified five early biosynthetic genes, namely two *CHS* genes (LOC101298162 and LOC101298456), one *F3H* gene (LOC101300182), and two *CHI* genes (LOC101305307 and -LOC101292744), that had up-regulated expression levels in BF1 and BF2*.* An earlier investigation indicated DFRs catalyze the conversion of colorless leucoanthocyanidins to colored anthocyanidins [39]. In the present study, the expression levels of genes encoding DFR (LOC101293459 and LOC101293749) were higher in BF1 and BF2 than in FN, resulting in the accumulation of anthocyanins in the two interspecific hybrids (Fig. 6). A recent study showed that high *DFR* expression levels lead to the production of colored anthocyanins in the Mitchell line of *Petunia* [32]. Similar findings were reported for *Litchi chinensis* Sonn [40] and *Malus hupehensis* [41]. In previous studies, increasing *DFR* expression and decreasing *FLS* expression led to increased anthocyanin contents and pink petals, with peak anthocyanin levels in *DFR*-sense and *FLS*-antisense transgenic lines [42, 43]. In this study, the expression of two *FLS* genes (LOC101309876 and LOC101309056) was significantly down-regulated in the two interspecific hybrids, which likely resulted in decreased quercetin contents and the increased conversion of dihydroquercetin to anthocyanin. In addition, anthocyanidin 3-O-glucosyltransferase (BZ1; EC:2.4.1.115) is the last key enzyme in the anthocyanin biosynthetic pathway. More specifically, it catalyzes the transfer of glucose from UDP-glucose to pelargonidin and cyanidin [44–46]. In the pale red fruits of BF1 and BF2, the expression levels of two *BZ1* genes (LOC101310867 and LOC101295404) were down-regulated, which was in contrast to the up-regulated expression of another *BZ1* gene (LOC101300000). The high *BZ1* (LOC101300000) expression level was consistent with the significant increase in the abundance of the downstream metabolites (e.g., pelargonidin 3-O-glucoside and pelargonidin-3-O-rutinoside) (Table 2, Fig. 6). The down-regulated expression of *BZ1* genes was in accordance with the decreased accumulation of the downstream metabolites [e.g., cyanidin-3-(6-O-p-caffeoyl)-glucoside, cyanidin-3-O-(6-O-p-coumaroyl)-glucoside, and cyanidin-3-O-sambubioside] (Table 2, Fig. 6). Therefore, we speculated that the up-regulated *BZ1* gene (LOC101300000) is the key gene regulating the synthesis of pelargonidin derivatives and the fruit coloration of the two interspecific hybrids. Considered together, these results indicate that the synergistic expression of specific genes (e.g., *CHS*, *CHI*, *F3H*, *DFR*, and *BZ1*) promotes the accumulation of anthocyanins in BF1 and BF2 fruits.

### Candidate transcription factor genes involved in anthocyanin biosynthesis

The enzymes catalyzing anthocyanin synthesis-related reactions in ornamental plants are encoded by genes that are regulated by TFs, including MYB, bHLH, and WD40 family members [47]. Many MYB TFs are considered to be potential key regulators of the anthocyanin biosynthetic pathway [48]. Furthermore, MYB TFs can function independently [49] or combine with other proteins to form a TF complex (MYB–bHLH–WD40) that regulates the biosynthesis of anthocyanins in a variety of plants, including banana [50], strawberry [20], and red pear (*Pyrus pyrifolia*) [47]. In the current study, 31 of the identified DEGs encoded TFs. To determine which TFs are involved in the fruit coloration of the two FN interspecific hybrids, we focused on the TFs strongly associated with five anthocyanin biosynthesis genes (two *CHS* genes, two *DFR* genes, and one *BZ1* gene) and two key pelargonidin derivatives (pelargonidin-3-O-glucoside and geranidin-3-O-rubutin). We determined that two key pelargonidin derivatives (geranidin-3-O-glucoside and geranidin-3-O-rubutin) and one *DFR* gene (LOC101293749) were significantly correlated with bHLH, MYB, WRKY, NAC, WD40, TCP, AP2, and bZIP TFs, whereas another *DFR* gene (LOC101293459) was not significantly correlated with WRKY, NAC, and TCP TFs. Two *CHS* genes (LOC101298162 and LOC101298456) and a *BZ1* gene (LOC101300000) were highly correlated with bHLH, WD40, and AP2 TF family members (Fig. 7). Accordingly, these TFs may co-regulate the expression of the structural genes involved in the anthocyanin biosynthesis in FN interspecific hybrids. The regulatory effects of MYB [6], WRKY [51], TCP [24], Bhlh [23], AP2 [52], and WD40[20] TFs on anthocyanin synthesis in strawberry have been reported.

## Conclusion

In the present study, we analyzed the fruits of FN and its interspecific hybrids BF1 and BF2 at the metabolome and transcriptome levels to identify the key anthocyanins, structural genes, and TFs modulating the fruit coloration of the two FN interspecific hybrids. Notably, pelargonidin-3-O-glucoside and pelargonidin-3-O-rutinoside may be the key pigments in the BF1 and BF2 fruits. Additionally, *DFR* (LOC101293459 and LOC101293749) and *BZ1* (LOC101300000) structural genes in the anthocyanidin biosynthetic pathway were significantly more highly expressed in BF1 and BF2 than in FN, suggesting that the encoded proteins may enhance the accumulation of two pelargonidin derivatives, thereby contributing to the formation of pale red fruit skin. Moreover, we identified *DFR* genes (LOC101293749 and LOC101293459) significantly correlated with bHLH, MYB, WD40, AP2, and bZIP TFs as well as a *BZ1* gene (LOC101300000) highly correlated with bHLH, WD40, and AP2 TF family members. The results of this study have elucidated the molecular regulation of anthocyanin biosynthesis and accumulation in FN and its interspecific hybrids and may be useful for the genetic improvement of strawberry varieties, leading to the production of fruits with more desirable colors.

## Materials and methods

### Fruit materials and sample collection

The plants of *Fragaria nilgerrensis* Schlecht (FN) in this experiment were collected from Wudang District, Guiyang City, Guizhou Province, China in 2017. Voucher specimens were deposited in the Herbarium in Guizhou Academy of Agricultural Sciences (GAAS, Yongbo Geng, 1,685,568,514@qq.com, voucher number: 201710005) and was identified as *F. nilgerrensis* by Professor Peilin Zhong. Two interspecific hybrids BF1 and BF2 were generated as follows:

$$\begin{array}{c}\mathrm{Benihoppe }(\mathrm{female}, 8x) \times Fragaria nilgerrensis\mathrm{ Schlecht}. (\mathrm{male}, 2x)\\ \downarrow \\ \begin{array}{c}5x\\ \downarrow \mathrm{ Chromosome doubling}\\ \begin{array}{c}\mathrm{BF }(\mathrm{female}, 10x)\\ \begin{array}{c}\downarrow \mathrm{ Seedling breeding}\\ \mathrm{BF}1\mathrm{ and BF}2\end{array}\end{array}\end{array}\end{array}$$

Briefly, the pentaploid progenies were generated from the distant hybridization between the strawberry cultivar ‘Benihoppe’ (with red fruit) and *F. nilgerrensis* (with white fruit). Decaploid seedlings were obtained after the chromosome doubling in the tips of the pentaploid stolons. BF1 and BF2 were obtained by seedling breeding of the decploid adult seedlings. All the plant materials were grown in the Strawberry Germplasm Repository of the Guizhou Horticulture Institute in Guiyang (26.492310 N, 106.653870 E), Guizhou province, China. Their accession code were GY-9, GY-1 and GY-20 for FN, BF1 and BF2, respectivly. In 2021, three biological replicates of ripe fruit samples were collected from FN (white skin; control) and its interspecific hybrids BF1 (pale red skin) and BF2 (pale red skin) and immediately frozen in liquid nitrogen and stored at − 80 °C for the subsequent RNA extraction and flavonoid content quantification.

### Analyses of the phenotype and color

The common L*, a*, and b* color space values were used to describe fruit colors. Specifically, at different equatorial positions of each fruit, L* (lightness), a* (green–red coordinate), and b* (blue–yellow coordinate) were measured using the NF555 spectrophotometer (Nippon Denshoku Industries Co., Ltd., Tokyo, Japan) and C* (saturation) (i.e., [(a*)^2^ + (b*)^2^]^0.5^) was calculated. Images of the fruits were used to compare phenotypes.

### Detection of flavonoids

To extract flavonoids, 0.5 mL methanol/water/hydrochloric acid (500,500:1, v/v/v) was added to 50 mg powdered fruit samples. The extract was vortexed for 5 min, ultrasonicated for 5 min, and centrifuged at 12,000 g for 3 min at 4 °C. After removing the supernatant, the residue was retained for a re-extraction using the above-mentioned procedure and conditions. The supernatants were passed through a 0.22 μm pore size membrane filter before being analyzed by UPLC-ESI–MS/MS (ExionLC™ AD UPLC system, https://sciex.com.cn/; Applied Biosystems 6500 Triple Quadrupole MS system, https://sciex.com.cn/). The analytical conditions were as follows: UPLC: column, Waters ACQUITY BEH C18 (1.7 μm, 2.1 mm × 100 mm); solvent system, water (0.1% formic acid), methanol (0.1% formic acid); gradient program, 95:5 v/v at 0 min, 50:50 v/v at 6 min, 5:95 v/v at 12 min, hold for 2 min, 95:5 v/v at 14 min, hold for 2 min; flow rate, 0.35 mL/min; temperature, 40 °C; injection volume, 2 μL.

Linear ion trap and triple quadrupole scans were acquired using a triple quadrupole-linear ion trap mass spectrometer (QTRAP® 6500 + LC–MS/MS system), equipped with an ESI Turbo Ion-Spray interface. The system was operated in the positive ion mode and controlled using the Analyst 1.6.3 software (Sciex). The ESI source operation parameters were as follows: ion source, ESI + ; source temperature, 550 °C; ion spray voltage, 5,500 V; curtain gas, 35 psi. Anthocyanins were analyzed via scheduled multiple reaction monitoring. Data were collected using the Analyst 1.6.3 software (Sciex). The Multiquant 3.0.3 software (Sciex) was used to quantify all metabolites. The chromatographic peaks of all metabolites were integrated and analyzed quantitatively using standard curves. Significantly differentially accumulated metabolites (DAMs) revealed by sample comparisons (BF1 *vs* FN and BF2 *vs* FN) were determined on the basis of the following criteria: |fold-change|> 2.0, and *P* < 0.05.

### Total RNA isolation and Illumina sequencing

Total RNA was extracted from the FN, BF1, and BF2 fruit samples using the TRIzol reagent (Thermo Fisher Scientific, Waltham, MA, USA). The RNA concentration and integrity were analyzed using the Qubit® RNA Assay Kit and the Qubit® 2.0 Fluorometer (Life Technologies, CA, USA) as well as the RNA Nano 6000 Assay Kit and the 2100 Bioanalyzer (Agilent Technologies, Palo Alto, CA, USA). Approximately, 1 μg RNA per sample was used as the input material for constructing sequencing libraries using the NEBNext® Ultra™ RNA Library Prep Kit for Illumina® (NEB, USA). The cDNA library quality was assessed using the 2100 Bioanalyzer (Agilent Technologies Inc.) and then sequenced using the Illumina HiSeq 2000 platform (Illumina, Inc., San Diego, CA, USA) to generate 125-bp/150-bp paired-end reads.

### RNA-seq data processing and DEG analysis

The fastp v0.19.3 program was used to filter the original data to obtain clean reads. The reference genome and its annotation files were downloaded from an online database (https://www.ncbi.nlm.nih.gov/genome/: search term = Fragaria + vesca + L.). The HISAT v2.1.0 program was used to construct the index and to compare the clean reads to the reference genome sequence. The StringTie v1.3.4d program was used for predicting new genes. StringTie applies network streaming algorithms and an optional de novo assembly algorithm to splice transcripts. The new genes were annotated according to the Kyoto Encyclopedia of Genes and Genomes (KEGG), Gene Ontology (GO), non-redundant (NR), Swiss-Prot, trEMBL, and EuKaryotic Orthologous Groups (KOG) databases using BLAST software. Additionally, featureCounts v1.6.2/StringTie v1.3.4d were used to align genes and calculate FPKM values, whereas DESeq2 v1.22.1/edgeR v3.24.3 were used to analyze the differential expression between two groups (BF1 *vs* FN and BF2 *vs* FN), with the P value corrected according to the Benjamini and Hochberg method. Significant differences in gene expression were determined on the basis of the following criteria: corrected P value < 0.05 and |log_2_(fold-change)|≥ 1. The KEGG pathway and GO term enrichment analyses of the DEGs were performed using the hypergeometric distribution test [52].

### Quantitative real-time PCR validation

The RNA samples used for the RNA-seq analysis were reverse transcribed into cDNA using the PrimeScript™ RT Reagent Kit (TaKaRa, Dalian, China). The obtained cDNA served as the template for the quantitative real-time polymerase chain reaction (qRT-PCR) analysis, which was completed using TB Green Premix Ex Taq™ II (TaKaRa) and the Bio-Rad CFX96 Real-Time PCR System (Bio-Rad Laboratories, Inc., USA). The qRT-PCR primer sequences are listed in Table S7. The PCR program was as follows: 95 °C for 3 min; 40 cycles of 95 °C for 10 s and 60 °C for 30 s.

### Statistical analysis

The flavonoid content and qRT-PCR analyses involved three biological replicates, with each biological replicate analyzed in triplicate. The flavonoid content data were analyzed using the DPS 7.05 software (China) and Microsoft Excel 2007. Significant differences were assessed by a one-way ANOVA followed by post hoc Duncan’s multiple range tests (*P* < 0.05). All data were expressed as the mean ± standard error. For the qRT-PCR analysis, *FaActin* was selected as the internal reference gene to determine relative DEG expression levels according to the comparative Ct (2^−ΔΔCt^) method [52, 55]. For the correlation analysis, the COR program from R was used to calculate Pearson’s correlation coefficient (PCC). The results of the corresponding correlation network analysis were visualized using the Cytoscape software v3.7.0. A PCC ≥ 0.8 and *P* ≤ 0.05 reflected a strong correlation.

## Supplementary Information

**Additional file 1.** **Table S1.** All108 metabolites identified from the FN vs BF1 and FN vs BF2 comparisons. **Table S2.** A total of 6967 co-significant DEGs were mapped into 109 KEGG database pathways. **Table S3.** Sixteen structural genes were predicted to be associated with anthocyanin biosynthesis. **Table S4.** 39 transcription factor DEGs were predicted to be associated with anthocyanin biosynthesis. **Table S5.** Analysis of the correlation between 39 transcription factor DEGs and 16 structural DEGs. **Table S6.** Analysis of the correlation between 39 transcription factor DEGs and 16 jointly up-regulated anthocyanins. **Table S7. **Primers used for qRT-PCR analysis.

## Data Availability

The datasets supporting the conclusions of this article are included within the article and its additional files. The raw data of RNA-seq that support the fndings of this study have been deposited in the NCBI (http://www.ncbi.nlm.nih.gov) database under accession code [BioProject ID PRJNA931590].

## Abbreviations

*FLS*: Flavonol synthase
*CHS*: Chalcone synthase
*F3H*: Naringenin 3-dioxygenase
*DFR*: Dihydroflavonol 4-reductase
*CHI*: Chalcone isomerase
*ANR*: Anthocyanidin reductase
*BZ1*: Anthocyanidin 3-O-glucosyltransferase
TFs: Transcription factors
DEG: Differentially expressed gene
DAM: Differentially accumulated metabolite
