# Supplementary material for: Transcriptomic analysis reveals the mechanism underlying the anthocyanin changes in Fragaria nilgerrensis Schlecht. and its interspecific hybrids
Source: BMC Plant Biol. 2023 Jul 11;23:356. doi: 10.1186/s12870-023-04361-1 (PMC10334533; doi:10.1186/s12870-023-04361-1)
Supplement: Supplementary file 1 — Additional file 1. Table S1. All108 metabolites identified from the FN vs BF1 and FN vs BF2 comparisons. Table S2. A total of 6967 co-significant DEGs were mapped into 109 KEGG database pathways. Table S3. Sixteen structural genes were predicted to be associated with anthocyanin biosynthesis. Table S4. 39 transcription factor DEGs were predicted to be associated with anthocyanin biosynthesis. Table S5. Analysis of the correlation between 39 transcription factor DEGs and 16 structural DEGs. Table S6. Analysis of the correlation between 39 transcription factor DEGs and 16 jointly up-regulated anthocyanins. Table S7. Primers used for qRT-PCR analysis. [file 12870_2023_4361_MOESM1_ESM.zip › Table S7.docx]

**Table S7** Primers used for qRT-PCR analysis.

| Gene | Forward primer (5′→ 3′) | Reverse primer (5′→ 3′) |
| --- | --- | --- |
| *FaActin* | CGAGCTGTTTTCCCTAGCAT | TCATCTTCTCACGATTAGCCTT |
| *gene-LOC101308793* | GATGCAGGCCAGTTGATGAC | TTGCAAGAGCAGTGAGGAGAAG |
| *gene-LOC101298456* | TACCGACTGGAACTCACTTTTC | TGCCTCGTGGCTTCTAACT |
| *gene-LOC101305307* | AAGGGAAGGAGGAGTCGAA | CCAAGCAGGATATGGTTGAA |
| *gene-LOC101309056* | GTTTGCTATTCCCATTGTCG | AGAAGCCCCATTCTTCACA |
| *gene-LOC101298162* | GTCTCAGCGGCCCAAACTAT | TCGAAATCAGCCCAGGAAC |
| *gene-LOC101307502* | CTGTTGTGCAGTTGCGTGTCA | CCTTCTTCAGGCCCTTCAATC |
| *gene-LOC101298756* | CTACGAAGTCGAGCATGATGTG | AGGTCGGTGGCGGAATAAA |
| *gene-LOC101295899* | CAGCAAGCAGTTCAAGGACAC | GGCCGCAGAATTTGTGATAGA |
